# Tumor neoantigens: from basic research to clinical applications

**DOI:** 10.1186/s13045-019-0787-5

**Published:** 2019-09-06

**Authors:** Tao Jiang, Tao Shi, Henghui Zhang, Jie Hu, Yuanlin Song, Jia Wei, Shengxiang Ren, Caicun Zhou

**Affiliations:** 1 Department of Pulmonary Medicine, Shanghai Respiratory Research Institute, Zhongshan Hospital, Fudan University, Shanghai, China; 2 The Comprehensive Cancer Centre of Drum Tower Hospital, Medical School of Nanjing University, No. 321, Zhongshan Road, Nanjing, 210008 China; 3 Beijing Genecast Biotechnology Co., Beijing, China; 4 Department of Medical Oncology, Shanghai Pulmonary Hospital & Thoracic Cancer Institute, Tongji University School of Medicine, No. 507, Zheng Min Road, Shanghai, 200433 China

**Keywords:** Neoantigen, Immunotherapy, Immune escape, Immune checkpoint, Resistance

## Abstract

Tumor neoantigen is the truly foreign protein and entirely absent from normal human organs/tissues. It could be specifically recognized by neoantigen-specific T cell receptors (TCRs) in the context of major histocompatibility complexes (MHCs) molecules. Emerging evidence has suggested that neoantigens play a critical role in tumor-specific T cell-mediated antitumor immune response and successful cancer immunotherapies. From a theoretical perspective, neoantigen is an ideal immunotherapy target because they are distinguished from germline and could be recognized as non-self by the host immune system. Neoantigen-based therapeutic personalized vaccines and adoptive T cell transfer have shown promising preliminary results. Furthermore, recent studies suggested the significant role of neoantigen in immune escape, immunoediting, and sensitivity to immune checkpoint inhibitors. In this review, we systematically summarize the recent advances of understanding and identification of tumor-specific neoantigens and its role on current cancer immunotherapies. We also discuss the ongoing development of strategies based on neoantigens and its future clinical applications.

## Introduction

Tumor neoantigen, or tumor-specific antigen (TSA), is the repertoire of peptides that displays on the tumor cell surface and could be specifically recognized by neoantigen-specific T cell receptors (TCRs) in the context of major histocompatibility complexes (MHCs) molecules [1–5]. From an immunological perspective, tumor neoantigen is the truly foreign protein and entirely absent from normal human organs/tissues. For most human tumors without a viral etiology, tumor neoantigens could derive from a variety of nonsynonymous genetic alterations including single-nucleotide variants (SNVs), insertions and deletions (indel), gene fusions, frameshift mutations, and structural variants (SVs) [2, 5]. For virally associated tumors, such as human papillomavirus (HPV)–related cervical or oropharyngeal cancer, Merkel cell polyomavirus (MCPyV)–related Merkel cell carcinoma (MCC) and Epstein-Barr virus (EBV)–related head and neck cancers, any epitopes derive from open reading frames (ORFs) in the viral genome also contribute to the potential source of neoantigens [6–8]. In recent years, emerging evidence has suggested that neoantigens play a pivotal role in tumor-specific T cell-mediated antitumor immunity. As summarized in several elegant reviews [2, 4, 5, 9], this evidence includes, but is not limited to, (1) the occurrence of antitumor immune response via T cell recognition of neoantigen, (2) the relationship between tumor mutation/neoantigen burden and clinical outcomes to immune checkpoint blockade, and (3) the promising antitumor effects of therapeutic vaccines or adoptive T cell transfer based on neoantigen.

Unlike neoantigens, another two common types of tumor antigens, named tumor-associated antigens (TAAs) and cancer-germline antigens (CGAs), are not only expressed on the tumor cell surface, they would also be found on healthy or immune-privileged tissues (especially reproductive tissues including testes, fetal ovaries, and trophoblasts) with low levels of expression [3, 10, 11]. Therapeutic vaccines based on the TAAs or CGAs have obtained dismal results mainly due to the central and peripheral tolerance mechanisms [12]. Moreover, high-affinity TCRs for TAAs are preferentially depleted because of positive selection, and the affinities of the remaining TCRs for TAAs are lower than those for neoantigens and other foreign antigens [13]. In addition, since TAAs or CGAs could still have low levels of expression in normal tissues, targeting them may result in severe autoimmune toxicities related to immune activation in non-target tissues, such as severe hepatitis, colitis, rapid respiratory failure, renal impairment, and even treatment-related death [14].

Theoretically, neoantigen is an ideal immunotherapy target because they are distinguished from germline and could be recognized as non-self by the host immune system [5]. Neoantigen-specific immune reactions are not easily subject to complex immune tolerance mechanisms. Additionally, it may be less likely to trigger autoimmunity because they do not express on normal cells. Neoantigen-based therapeutic personalized vaccines and adoptive T cell transfer have shown promising preliminary results. Furthermore, recent studies suggested the significant role of neoantigen in immune escape, immunoediting, and sensitivity to immune checkpoint inhibitors.

In this review, we systematically summarize the recent advances of understanding and identification of tumor-specific neoantigens and its role on current cancer immunotherapies. We also discuss the ongoing development of strategies based on neoantigens and its future clinical applications. We do hope that this review could help us better understand the ongoing development of strategies based on neoantigens and its future clinical applications.

## Historical overview in the understanding of tumor neoantigens

**Fig. 1:**
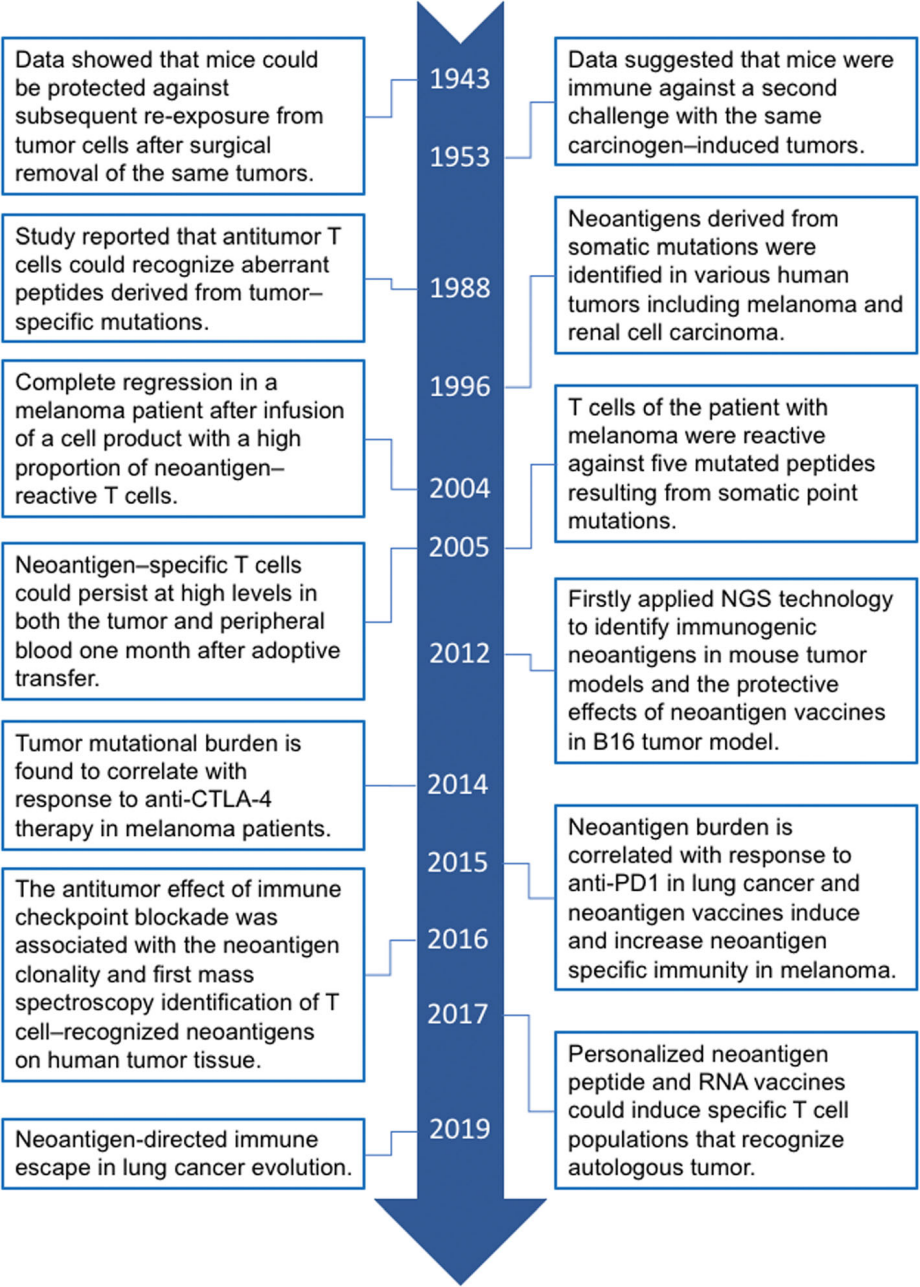
Historical overview in understanding of tumor neoantigens

Currently, it is well-known that the immune system possesses an extraordinary ability to distinguish self from non-self, and recognize and target non-self antigens on abnormal cells [4]. However, our understanding of tumor neoantigens and its important role in antitumor immune response is a long and tortuous process (Fig. 1). We can go back to the late nineteenth century when William Coley, father of cancer immunotherapy, firstly attempted to leverage the patient’s immune system to treat cancer [15, 16]. In spite of the tremendous outcomes in individual cases, his findings were abandoned due to the huge success of chemotherapy and radiotherapy in control of various cancers. The investigation of the immune system in carcinogenesis and control of tumor growth during progression has recurred in the early part of the twentieth century [17]. In 1943, Gross et al. firstly showed that mice could be protected against subsequent re-exposure from tumor cells after surgical removal of the same tumors [18]. They also found that analogous protection against tumor cells could be induced by first exposing mice to lethally irradiated tumor cells. Their findings revealed that the immune system can recognize and eliminate malignant cells. Ten years later, another group further found that mice were immune against a second challenge with the same tumor cells after resection of carcinogen-induced tumors, supporting the idea of the existence of antitumor immunity [19]. Nevertheless, the nature of antigens that could substantially trigger antitumor immune response was unclear during this period. Several decades later, De Plaen and colleagues reported a significant finding that antitumor T cells could recognize aberrant peptides derived from tumor-specific mutations in a methylcholanthrene (MCA)-induced mouse tumor model [20]. They further identified the first T cell–recognized neoantigen by using a cDNA library screen. After that, a series of neoantigens derived from somatic mutations were identified in various human tumors including melanoma and renal cell carcinoma [21, 22]. Another significant advance in our understanding of neoantigens occurred in the early twenty-first century, when Huang et al. found nearly complete regression in a melanoma patient after infusion of a cell product with a high proportion of neoantigen-reactive T cells [23] and Lennerz et al. reported that the T cells of the patient with melanoma were reactive against five mutated peptides resulting from somatic point mutations and T cells against mutated epitopes was clearly predominated over the response to TAAs [24]. Similarly, the Rosenberg group found that neoantigen-specific T cells could persist at high levels in both the tumor and peripheral blood 1 month after adoptive transfer in a patient with melanoma that experienced a complete regression following adoptive transfer of ex vivo–expanded autologous tumor-reactive tumor-infiltrating lymphocytes (TILs)[25]. Collectively, these studies provided initial evidence that neoantigens play a vital role in the naturally occurring antitumor T cell response. With the advent and wide application of next-generation sequencing (NGS) technology, we have obtained a more and more profound understanding on tumor neoantigens. In 2012, two research groups independently and firstly applied NGS technology to identify immunogenic neoantigens in mouse tumor models [26, 27] and reported the protective effects of neoantigen vaccines in B16 tumor model [27]. Soon after, NGS technology was widely applied to explore T cell recognition of neoantigens in cancer patients [28]. Subsequent studies indicated that T cell responses against mutated antigens are frequently observed within TIL products in types of cancers including melanoma, lung cancer, colorectal cancer, cholangiocarcinoma, and squamous cell carcinoma of the head and neck [29–33]. Nonetheless, whether the fraction of patients with detectable neoantigen-specific T cell responses is comparable across these tumor types remains largely unknown at this moment. Recent advance in our understanding of neoantigens is derived from the research of immune checkpoint inhibitors targeting cytotoxic T lymphocyte-associated protein 4 (CTLA-4) and programmed cell death protein 1 (PD-1) on T cells. The cancer-immunity cycle indicates that T cells recognized neoantigens displayed by MHCs on tumor cells is the first step to eliminate the established tumor via using immune checkpoint inhibitors [34, 35]. Mechanically speaking, there should be a significant correlation between tumor neoantigen load and clinical efficacy of immune checkpoint inhibitors. This relationship was also demonstrated by several elegant studies [33, 36–38]. However, some cancers with high level of neoantigen load are not as sensitive to immune checkpoint inhibitors as expected. It is likely to be associated with the neoantigen clonality [39] but true mechanism of this phenomenon is not fully understood, suggesting that we still need to pay more effort to deepen our understanding of tumor neoantigens and its role in antitumor immune response.

## Prediction and identification of tumor-specific neoantigens

The genetic changes harbored by a neoantigen could transform a self-protein into a functionally non-self-protein. The neoepitopes being presented by MHC-I could then drive immunogenic responses through several potential mechanisms. First, neoepitopes derived from mutations could improve MHC-I binding affinity and then result in the presentation of MHC-I ligands that would ordinarily not be presented during T cell selection [40]. Second, neoepitopes may increase the stability of the TCR–MHC-I interaction even with similar binding affinities as wild-epitopes and can induce different immune responses [41]. Third, mutation-induced changes to flanking amino acid residues significantly interfere the presentation of MHC-I viral epitopes. Theoretically, a mutation harbored within a neoantigen could drive the presentation of an adjacent unmutated MHC-I ligand that has escaped immune tolerance due to poor processing [42, 43].

**Fig. 2:**
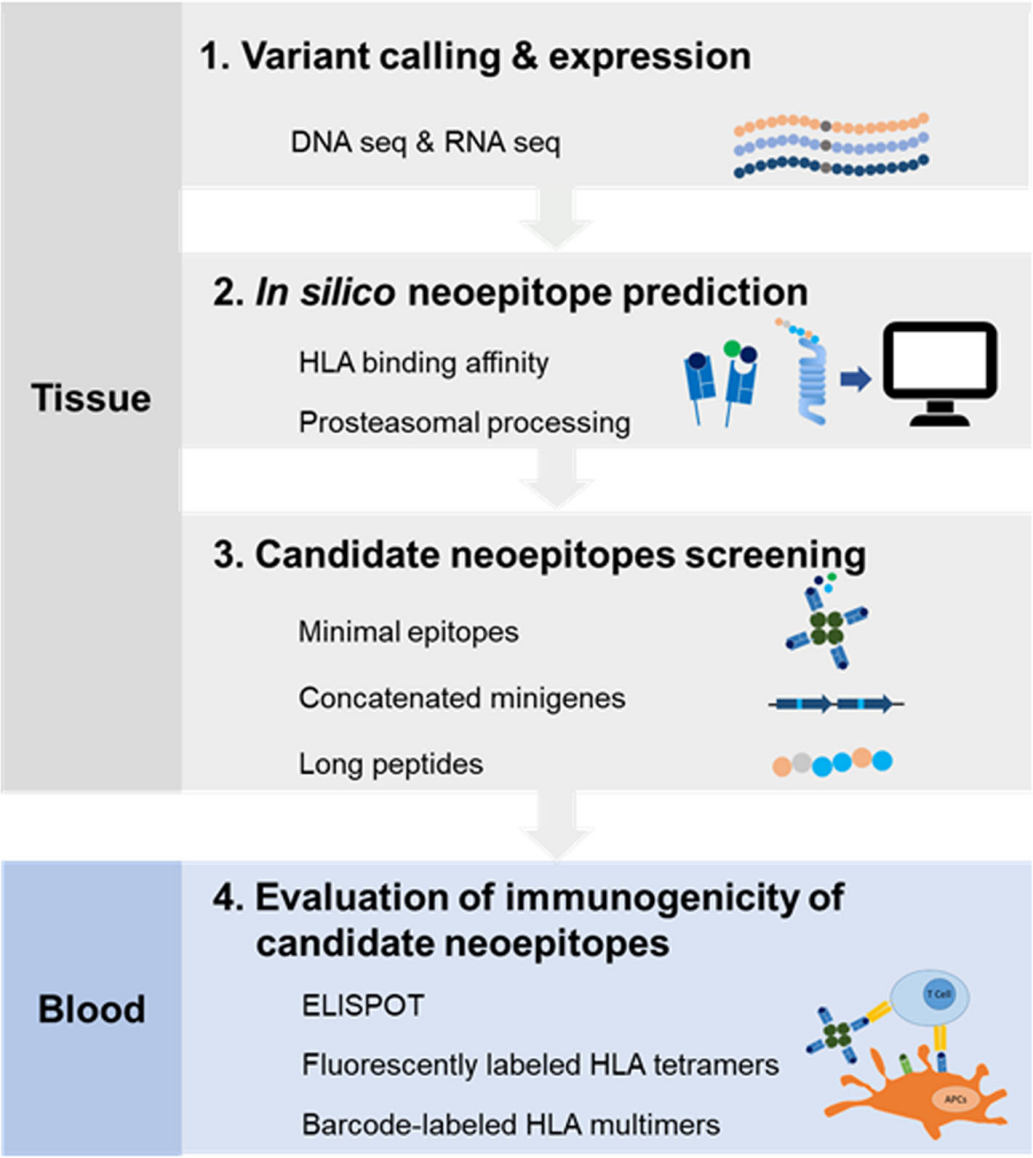
Flowchart for tumor neoantigen prediction and detection of T cell–recognized neoantigens

Neoepitopes can be identified in various ways. Prior to the advent of massively parallel NGS, labor-intensive individual cDNA library screening was used to screening T cell–reactive neoepitopes [24]. When NGS became a routine technique, the ability to identify tumor-specific genetic mutations altering the protein coding regions became rapid and high throughput, facilitating neoantigen prediction. This is accomplished by applying machine learning algorithms that model aspects of the MHC-I processing and presentation pathway to patient tumor exome data to predict potential neoepitope targets [44, 45]. Predicted neopeptides can next be synthesized and tested for reactivity by autologous T cells using various assays such as ELISPOT, fluorescently labeled HLA tetramers, or barcode-labeled HLA multimers [46]. A classic procedure for identification of neoepitopes was shown in Fig. 2.

Several machine learning–based epitope prediction tools are available, such as NetMHCpan [47], NetMHCIIpan [48], MHCflurry [49], ConvMHC [50], PLAtEAU [51], and NetCTLpan [52]. For a neopeptide to become a neoepitope, two properties must be fulfilled: the peptide must be processed and presented by HLA, and the presented peptide must be recognized by a suitable T cell. Therefore, although these approaches show immense potential, current neoepitope prediction methods based on sequencing and predictions of epitope processing and presentation result in a low rate of validation. Bjerregaard et al. summarized published data from 13 publications on human neopeptides originating from single amino acid substitutions for which T cell reactivity had been experimentally tested. Less than 3% were reported to elicit the T cell response [53]. One major reason may be that the machine learning algorithms are highly dependent on the datasets available for training and testing [54]. As a widely used resource, The Immune Epitope Database and Analysis Resource (IEDB) hosts a database of experimentally validated epitopes. But its datasets of validated T cell epitopes found in databases are almost entirely formed of epitopes from bacteria or viruses and were not obtained by standardized experimental methodologies in cancer models [55].

Recent developments in HLA peptidomics for class I and II HLA molecules have been relevant for the improvements in the available epitope datasets [56, 57]. Abelin et al. set up their own dataset (> 24,000 peptides) and identified thousands of peptides bound to 16 different HLA class I alleles to quantify the contribution of factors critical to epitope presentation, such as protein cleavage and gene expression [58]. A commercial platform, EDGE (Epitope Discovery in cancer GEnomes) was constructed on deep learning to a large (*N* = 74 patients) HLA peptide and genomic dataset from various human tumors and could increase the positive predictive value of HLA antigen prediction by up to nine-fold [59]. Another strategy to improve the ability to predict neoepitope was the integration of potential immunogenicity assessments to the prediction process. MuPeXI algorithm ranks predicted neoepitopes by a priority score that is based on inferred abundance, MHC binding affinity, and an immunogenicity score based on similarity to non-mutated wild-type peptide [60] EpitopeHunter algorithm, which integrates RNA expression with immunogenicity prediction algorithm based on the hydrophobicity of the TCR contact region [61, 62]. Neopepsee algorithm using a machine learning algorithm trained on epitope features, including antigen processing and presentation, amino acid characteristics, the binding difference between wild-type and mutant epitope, and similarity to known epitopes, to predict the immunogenicity and reduce the false-positive rate [63]. In conclusion, to maximizing the probability of identifying clinically relevant neoepitopes, multiple method epitope prediction and advanced neoepitope quality metrics are warranted.

## Neoantigens-directed immunoediting and immune escape

Cancer immunoediting is a conceptual framework integrating the immune system’s dual host-protective and tumor-promoting roles. During cancer immunoediting, the host immune system shapes tumor fate in three phases: elimination, equilibrium, and escape [64]. Decades of researches have revealed the dual role of the immune system in tumorigenesis. Recent work on cellular or animal model and clinical study on one cancer patient case [26, 64–67] have shown unequivocal evidence that the immune system can facilitate cellular transformation, prevent or control tumor outgrowth and shape the immunogenicity of tumors [68–70]. Studies using tumor exome sequencing to predict candidate neoantigens provide insights into the contribution of mutated peptide antigens encoded by somatic mutations to tumor antigenicity in human cancers [28, 71].

Interactions between the immune system and tumors have clear functional significance for tumor control, as the immune system exerts evolutionary pressure on highly immunogenic tumor clones through the process of immunoediting [26, 65, 72], and antitumor immune responses can be enhanced therapeutically by agents such as immune checkpoint inhibitors [73]. However, until recently, several multi-omics studies demonstrated the direct impact from immune pressure and immune editing on clonal evolution of tumor cells in metastases, benefit from the development of bioinformatic approaches on neoantigen prediction and on tumor evolutional and metastasis model based on somatic mutational landscape from lesions from different space and time [74–79]. A total of 258 samples from different regions of 88 early stage, treatment-naïve NSCLC were examined in the Tracking Cancer Evolution through Therapy (TRACERx) NSCLC study [77]. Similar to genomic heterogeneity, the immunological landscapes of different regions of the same tumor can vary dramatically. An increase in the ratio of observed-to-expected neoantigens was noted from clonal to subclonal mutations among tumors with a low level of immune cell infiltration, which possibly reflects an ancestral immune-active microenvironment that has subsequently become cold. Tumors with high or heterogeneous levels of immune cell infiltration had significantly lower levels of expressed clonal neoantigens than those with limited levels of infiltration (median 29% and 35% versus 41%; *P* < 0.01). The pattern of neoantigen quantity and/or quality changes reflecting the immunoediting was also observed in other solid tumors including pancreatic cancer [79], colorectal cancer [74], melanoma [75], and glioblastomas [78].

Tumor cells could evolve several mechanisms to escape immune responses. On the one hand, cancers can hijack mechanisms developed to limit inflammatory and immune responses and escape the immuno-elimination. On the other hand, the metabolic or genetic alterations of tumor cells can render themselves invisible to the immune system or can favor the generation of an extracellular milieu preventing immune cell infiltration or cytotoxicity. And the tumor clone possessing neoantigens with potent immunogenicity tends to be eliminated during immunoediting. There are several ways through which immunogenic neoantigens could escape from immunological surveillance. At the DNA level, chromosomal instability–induced copy number alterations may drive the loss of neoantigens [77]. At the RNA level, the neoantigen expression could be decreased for the promoter hypermethylation, while epigenetics mechanism could not account for all the transcriptomic neoantigen depletion. Additional mechanisms of neoantigen transcription repression need elucidation [76, 77, 80]. At the protein level, the machinery to presenting antigen peptides could be disrupted by mutations affecting HLA heterozygosity, MHC stability, HLA enhanceosome, or neopeptide generation [77, 81]. All these different mechanisms through which the tumor hiding target to evade immune predation provide series potential clinical setting worth to be exploited [82].

## Preclinical and clinical neoantigen-based studies

Recent preclinical and clinical studies have shown that neoantigen-based approaches are able to induce robust antitumor immune responses in individual tumor microenvironment (TME). The two main approaches targeting tumor neoantigen that are well-established now include neoantigen-based cancer vaccines and neoantigen-based adoptive cell transfer (ACT) treatment. Also, combinational therapies employing both neoantigen-based approaches and immune checkpoint blockade (ICB) are underway to overcome ICB-induced immune resistance and maximize antitumor immune activity [83].

### Neoantigen-based cancer vaccines

Personalized vaccines targeting neoantigens are designed to prime and amplify neoantigen-specific T cell populations in vivo to augment adoptive antitumor immunity among individuals. Actually, cancer vaccines were first employed to target TAAs, which are overexpressed in tumors but also expressed in normal tissues [84]. However, previous clinical trials of TAA-based cancer vaccines failed to demonstrate durable and effective beneficial efficacy due to the deficient T cell priming in TME [85]. In contrast, neoantigens detected via NGS or mass spectrometry could result in decreased systematic immune tolerance and improved safety profile [56]. Thus, enthusiasm on neoantigen-based vaccines is increasing rapidly, with several recent preclinical and clinical studies demonstrating its potent activation of antitumor immune responses.

Recent preclinical studies have shown the efficacy and feasibility of neoantigen-targeted cancer vaccines on murine tumor models among melanoma [86–88], colon carcinoma [89], esophageal squamous cell carcinoma [90], sarcoma [91], and glioma [92]. Theresa et al. reported the potential role of IDH1 (R132H) mutation–specific vaccination for glioma treatment. They synthesized neopeptides containing IDH1 (R132H) p123-142 mutated region to bind to transgenic human MHC-II molecules in glioma mouse model. Results after vaccination indicated that neopeptide vaccine lead to effective mutation-specific antitumor immune responses in the mouse model with IDH1 (R132H)-mutated gliomas [92]. In addition, a synthetic DNA vaccine targeting multi-neoantigens reported by Elizabeth et al. also showed the potent ability of immune activation. The electroporation-mediated DNA vaccine delivery in this research was proved to be immunogenic and induced predominantly MHC-I restricted CD8^+^ T cell responses in C57BL/6 mice, resulting in the direct killing of tumor cells by the expanded neoantigen-specific T cells [88]. Thus, the DNA platform may have unique advantages to prime T cell activation for neoantigen-based vaccines.

The encouraging results from preclinical studies have also accelerated the development of clinical trials of neoantigen-based vaccines [93–98]. The first reported phase I clinical trial of neoantigen-based vaccines was from Beatriz’s team in 2015 [93]. They found that dendritic cell (DC) vaccine could promote the presentation of neoantigens by HLA-A*02:01 in three patients with advanced melanoma. Soon after, two significant studies published by Ott et al. and Sahin et al. in 2017 confirmed the potent role of neoantigen-based vaccines in melanoma treatment [96, 97]. Ott et al. employed a synthetic long peptide (SLP) vaccine targeting up to 20 predicted personal tumor neoantigens into six melanoma patients, among whom four patients did not have tumor recurrence within 25 months after vaccination and two other patients with recurrence achieved complete tumor regression (CR) when they received PD-1 antibodies [96]. Sahin et al. generated the first personalized RNA mutanome vaccines for melanoma treatment. Of the 13 patients, a third of patients achieved CR to RNA vaccination combined with PD-1 blockade therapy and detected enhanced neoantigen-specific T cell priming in vivo [97]. Apart from melanoma, most recently two co-published works in *Nature* expanded the potential role of neoantigen-based vaccines in the treatment of human glioblastoma [94, 95]. In a phase I study reported by Keskin et al., eight enrolled glioblastoma patients with multi-epitope neoantigen vaccination presented an increased number of neoantigen-specific CD4^+^ and CD8^+^ TILs [95]. In addition, another phase I trial showed that personalized neoepitope vaccine (APVAC2) induced predominantly CD4^+^ Th1 cell responses among 15 glioblastoma patients [94]. These findings demonstrated that neoantigen-based vaccines were feasible for “cold” tumors such as glioblastoma, which commonly have low mutation load and immunosuppressive TME. To date, several phase I/II clinical trials of neoantigen-based cancer vaccines are underway among various types of cancers (Table 1). Collectively, neoantigen vaccines based on DNA, RNA, SLP, and DC have shown promising results of neoantigen-specific T cell infiltration and responses both in preclinical and clinical studies.

**Table 1:** Current clinical trials of neoantigen-based cancer vaccines

Interventions	NCT number	Phase	Enrollment status	Cancer types	Combinations
Neoantigen vaccine	NCT03558945	I	Recruiting	Pancreatic tumor	None
Neoantigen vaccine	NCT03359239	I	Recruiting	Urothelial/bladder cancer	Atezolizumab
Neoantigen vaccine	NCT03645148	I	Recruiting	Pancreatic cancer	GM-CSF
Peptide vaccine	NCT03558945	II	Not yet recruiting	TNBC	Nab-paclitaxel, Durvalumab
Peptide vaccine	NCT03929029	I	Not yet recruiting	Melanoma	Nivolumab, ipilimumab
Peptide vaccine	NCT03715985	I	Recruiting	Solid tumors	None
Peptide vaccine	NCT01970358	I	Active, not recruiting	Melanoma	None
Peptide vaccine	NCT03639714	I/II	Recruiting	Solid tumors	Nivolumab, ipilimumab
Peptide vaccine	NCT03956056	I	Not yet recruiting	Pancreatic cancer	Adjuvant chemotherapy
Peptide vaccine	NCT02287428	I	Active, not recruiting	Glioblastoma	Radiation therapy
Peptide vaccine	NCT02950766	I	Recruiting	Kidney cancer	Ipilimumab
Peptide vaccine	NCT03219450	I	Not yet recruiting	Lymphocytic leukemia	Cyclophosphamide
Peptide vaccine	NCT03422094	I	Recruiting	Glioblastoma	Nivolumab, ipilimumab
DC vaccine	NCT03871205	I	Not yet recruiting	Lung cancer	None
DC vaccine	NCT02956551	I	Recruiting	NSCLC	None
DC vaccine	NCT03674073	I	Recruiting	Hepatocellular carcinoma	Microwave ablation
DC vaccine	NCT03300843	II	Recruiting	Solid tumors	None
RNA vaccine	NCT03908671	Not Applicable	Not yet recruiting	Esophageal cancer, NSCLC	None
RNA vaccine	NCT03480152	I/II	Recruiting	Solid tumors	None
RNA vaccine	NCT03468244	Not Applicable	Recruiting	Solid tumors	None
DNA vaccine	NCT03532217	I	Recruiting	Prostate cancer	Nivolumab, Ipilimumab
DNA vaccine	NCT03122106	I	Recruiting	Pancreatic cancer	Adjuvant chemotherapy
DNA vaccine	NCT03199040	I	Recruiting	TNBC	Durvalumab

*GM-CSF* granulocyte-macrophage colony stimulating factor, *TNBC* triple negative breast cancer, *NSCLC* non-small cell lung cancer, *DC* dendritic cells

### Neoantigen-based adoptive T cell transfer

As an alternative to neoantigen vaccines, the neoantigen-based ACT approaches treat patients with T cell products manufactured in vitro that contain abundant group of neoantigen-specific T cells [2]. Compared to neoantigen-based vaccines, neoantigen-based ACT therapies have several potential advantages including the higher population of neoantigen-reactive T cells (NRTs) and the less immunosuppressive effects from TME during the amplification phase of NRTs.

Since the first report from Rosenberg group in 2015 that neoepitopes derived from somatic mutations in common gastrointestinal cancers could be immunogenic for personalized TILs recognition [99], several studies have confirmed that neoantigens derived from immunogenic mutations induced neoantigen-specific T cell activation among lung cancer, head and neck squamous cell carcinoma, colorectal cancer, breast cancer, and lymphoma [100–105]. However, TILs could easily acquire a dysfunctional state and result in very modest replicative capacity and immune responses. Thus, it may be more advantageous to transduce neoantigen-specific TCR sequences into patients’ peripheral blood lymphocytes (PBLs). Data from a preclinical study proved that TCR-engineered PBLs reactive with KRAS mutant neopeptides reduced xenograft mouse model of melanoma and other cancers, supporting the feasibility of TCR-T therapy [106].

As to clinical studies of neoantigen-based ACT therapies, the Rosenberg group has successfully treated patients with this strategy in melanoma and other malignancies [30, 107–110]. They first treated a patient with metastatic epithelial cancer via the adoptive transfer of ERBB2 interacting protein (ERBB2IP) mutation–reactive CD4^+^ TILs to achieve tumor regression in 2014 [110]. After that, Rosenberg et al. also identified TILs and memory T cells from patient peripheral blood which recognized KRAS- and TP53-mutated neoepitopes to treat patients with epithelial cancers [108, 109]. Additionally, they observed the promising results from clinical trials of metastatic colorectal and breast cancer. Complete and durable tumor regressions were observed in patients with breast cancer treated with four neoantigen (SLC3A2, KIAA0368, CADPS2, and CTSB)-reactive TILs and in a colorectal patient treated with mutant KRAS G12D reactive CD8^+^ TILs [30, 107]. All these works supported a significant role of neoantigen-specific T cells in cancer immunotherapies. The treatment applications and effects of neoantigen-reactive TILs could be expanded and enhanced through transduction of specific TCR sequences into other naïve CD8^+^ T cells to manufacture neoantigen-specific TCR-T cells for patients with the same mutations. More recently, our group constructed an inventory-shared neoantigen peptide library of common solid tumors to match the hotspot somatic mutations. Six of 13 patients using this novel neoantigen identification strategy achieved tumor regression. One metastatic thymoma patient achieved CR more than 29 months after NRT treatment [111]. Generally, developments of efficient and timely procedures to identify and amplify neoantigen-specific T cells also play an important role for augmenting personized neoantigen-based immunotherapies.

### Combinational therapies

Neoantigen-based vaccine and ACT treatment showed promising results in preliminary investigations, but they still need to combine with other therapeutic strategies to further enhance the antitumor effect due to the inevitably immunosuppressive effect of inhibitory immune checkpoints in TME [9, 112, 113]. As we summarized in Table 1, current combinational therapies could be roughly divided into the combination with other immunotherapies and other conventional therapies. Since PD-1/PD-L1 pathway exerts immunosuppressive effects on CD8^+^ T cells mediate antitumor immunity, neoantigen-based vaccine and ACT treatment plus anti-PD-1/PD-L1 antibodies could yield a strong antitumor immune response. In the preliminary studies, two research teams independently demonstrated that PD-1/PD-L1 blockade could markedly improve neoantigen-based vaccine-induced immune response [96, 97]. Specifically, Sahin et al. observed the long-lasting complete response in a patient treated with neoantigen-based vaccine plus PD-1 blockade therapy [97]. Their results suggested that it is valuable to test the efficacy of neoantigen-based vaccine or ACT in combination with checkpoint blockade and other immunotherapies. Theoretically, some conventional therapies could result in the release of tumor-associated antigens and neoantigens [114–117], suggesting the possibility for synergy with neoantigen-based vaccine therapy. Moreover, some chemotherapeutic drugs have been shown to augment the antigenicity and immunogenicity of tumor cells via altering neoantigen repertoire, increasing antigen production, improving antigen presentation, and augmenting T cell trafficking and responses [116–119]. Herein, we should not simply consider chemotherapy as tumor suppressive but treat it as the positive modulation of the immune system [120]. Several phase I trials are ongoing to investigate the safety and efficacy of neoantigen vaccine therapy plus chemotherapy in adjuvant setting (Table 1). These results are eagerly anticipated.

### Biomarker for immune checkpoint inhibitors

Immune checkpoint blockade has shown significant therapeutic responses against tumors containing high tumor mutational burden (TMB) or tumor neoantigen burden (TNB) [2, 33, 37]. A series of clinical trials reported that patients with NSCLC or melanoma had high objective responses to immune checkpoint blockade. Both two tumor types have the highest somatic mutation burdens among common solid tumors [121, 122]. In contrast, cancers with low TMB/TNB such as prostate cancer have shown little benefit from immune checkpoint inhibitors. These results provided additional evidence for the significance of neoantigens in the antitumor immune response. Notably, it was not the linear association between TNB and immune checkpoint inhibitors response. For example, some cases with high neoantigen burden showed no response to immune checkpoint therapies, as well as some with low neoantigen load, were susceptible to immune checkpoint inhibitors [123]. Interestingly, if we put two main factors including the likelihood of neoantigen presentation by the MHC and subsequent recognition by T cells into a neoantigen fitness model, we observed that this model could better predict survival in anti-CTLA-4-treated patients with melanoma and anti-PD-1-treated patients with lung cancer [124]. In addition, neoantigens may need to be expressed in every tumor cell in order to be efficiently targeted (so-called clonal neoantigen). An interesting study by McGranahan et al. reported that the therapeutic effect of immune checkpoint inhibitors was enhanced in NSCLC and melanoma enriched for clonal neoantigens, suggesting the significant role of neoantigen heterogeneity in antitumor immunity and supporting therapeutic developments targeting clonal neoantigens [39]. Taken together, these results revealed that high TNB represents merely a higher likelihood of the presence of immunogenic neoantigen, suggesting that neoantigen landscape alone is insufficient in predicting immune checkpoint inhibitor responses.

### Resistance mechanism for immune checkpoint inhibitors

Considering the significant role of tumor neoantigens in response to immune checkpoint inhibitors, it is reasonable to suppose that the evolution of neoantigen landscape would mediate resistance to immune checkpoint inhibitors. A retrospective study included 42 patients with NSCLC treated with PD-1 antibody monotherapy or combined PD-1 and CTLA-4 blockade and examined the evolving neoantigen landscape during the emergence of acquired resistance [73]. Among four consecutive cases that developed acquired resistance, the authors found that neoantigen loss via elimination of tumor subclones or via deletion of chromosomal regions containing truncal alterations, was associated with changes in T cell receptor clonality, then resulting in acquired resistance to immune checkpoint blockade. These results imply that the dynamics of neoantigen loss may be one of potential resistance mechanism. Widening the breadth of neoantigen reactivity may delay the development of acquired resistance to immune checkpoint blockade. However, it should be pointed out that this study had several limitations including small sample size, possibility of tumor heterogeneity, and analysis of samples from a defined period of relatively early acquired therapeutic resistance. Future investigations with a larger number of patients that developed acquired resistance to immune checkpoint inhibitors are still needed.

## Conclusions

In conclusion, emerging evidence suggests that tumor neoantigen plays a pivotal role in immune escape, antitumor immune response and successful cancer immunotherapies. Both neoantigen-based vaccine and ACT treatments show very promising antitumor effect together with high specificity and safety in preliminary studies. Combinatorial approaches of neoantigen-based therapies with other immunotherapies (e.g., immune checkpoint inhibitors) and conventional treatments are ongoing, and the results are eagerly anticipated. With a better understanding of biological properties and role of neoantigen in antitumor immunity, there are abundant reasons to believe that neoantigen-based therapeutic strategies have a bright future in cancer immunotherapies.

## Data Availability

Not applicable

## Abbreviations

ACT: Adoptive cell transfer
CGAs: Cancer-germline antigens
CTLA-4: Cytotoxic T lymphocyte-associated protein 4
DC: Dendritic cell
EBV: Epstein-Barr virus
HPV: Human papillomavirus
ICB: Immune checkpoint blockade
IEDB: Immune Epitope Database and Analysis Resource
indel: Insertions and deletions
MCA: Methylcholanthrene
MCC: Merkel cell carcinoma
MCPyV: Merkel cell polyomavirus
MHCs: Major histocompatibility complexes
NGS: Next-generation sequencing
ORFs: Open reading frames
PBLs: Peripheral blood lymphocytes
PD-1: Programmed cell death protein 1
SLP: Synthetic long peptide
SNVs: Single-nucleotide variants
SVs: Structural variants
TAAs: Tumor-associated antigens
TCRs: T cell receptors
TILs: Tumor-infiltrating lymphocytes
TMB: Tumor mutational burden
TME: Tumor microenvironment
TNB: Tumor neoantigen burden
TSA: Tumor-specific antigen

## References

[1] Gilboa E (1999). The makings of a tumor rejection antigen. Immunity.

[2] Schumacher TN, Schreiber RD (2015). Neoantigens in cancer immunotherapy. Science.

[3] Ward JP, Gubin MM, Schreiber RD (2016). The role of neoantigens in naturally occurring and therapeutically induced immune responses to cancer. Adv Immunol.

[4] Yarchoan M, Johnson BA, Lutz ER, Laheru DA, Jaffee EM (2017). Targeting neoantigens to augment antitumour immunity. Nat Rev Cancer.

[5] Schumacher TN, Scheper W, Kvistborg P (2019). Cancer neoantigens. Annu Rev Immunol.

[6] Walboomers JM, Jacobs MV, Manos MM, Bosch FX, Kummer JA, Shah KV, Snijders PJ, Peto J, Meijer CJ, Munoz N (1999). Human papillomavirus is a necessary cause of invasive cervical cancer worldwide. J Pathol.

[7] Gillison ML, Koch WM, Capone RB, Spafford M, Westra WH, Wu L, Zahurak ML, Daniel RW, Viglione M, Symer DE, Shah KV, Sidransky D (2000). Evidence for a causal association between human papillomavirus and a subset of head and neck cancers. J Natl Cancer Inst.

[8] Feng H, Shuda M, Chang Y, Moore PS (2008). Clonal integration of a polyomavirus in human Merkel cell carcinoma. Science.

[9] Chu Y, Liu Q, Wei J, Liu B (2018). Personalized cancer neoantigen vaccines come of age. Theranostics.

[10] Simpson AJ, Caballero OL, Jungbluth A, Chen YT, Old LJ (2005). Cancer/testis antigens, gametogenesis and cancer. Nat Rev Cancer.

[11] Kalejs M, Erenpreisa J (2005). Cancer/testis antigens and gametogenesis: a review and “brain-storming” session. Cancer Cell Int.

[12] Coulie PG, Van den Eynde BJ, van der Bruggen P, Boon T (2014). Tumour antigens recognized by T lymphocytes: at the core of cancer immunotherapy. Nat Rev Cancer.

[13] Stone JD, Harris DT, Kranz DM (2015). TCR affinity for p/MHC formed by tumor antigens that are self-proteins: impact on efficacy and toxicity. Curr Opin Immunol.

[14] Pan RY, Chung WH, Chu MT, Chen SJ, Chen HC, Zheng L, Hung SI (2018). Recent development and clinical application of cancer vaccine: targeting neoantigens. J Immunol Res.

[15] Coley WB. The treatment of malignant tumors by repeated inoculations of erysipelas. With a report of ten original cases. 1893. Clin Orthop Relat Res. 1991:3–11. 1984929

[16] Starnes CO (1992). Coley’s toxins in perspective. Nature.

[17] Waldmann TA (2003). Immunotherapy: past, present and future. Nat Med.

[18] Gross L (1943). Intradermal immunization of C3H mice against a sarcoma that originated in an animal of the same line. Cancer Res.

[19] Foley EJ (1953). Antigenic properties of methylcholanthrene-induced tumors in mice of the strain of origin. Cancer Res.

[20] De Plaen E, Lurquin C, Van Pel A, Mariame B, Szikora JP, Wolfel T, Sibille C, Chomez P, Boon T (1988). Immunogenic (tum-) variants of mouse tumor P815: cloning of the gene of tum- antigen P91A and identification of the tum- mutation. Proc Natl Acad Sci U S A.

[21] Robbins PF, El-Gamil M, Li YF, Kawakami Y, Loftus D, Appella E, Rosenberg SA (1996). A mutated beta-catenin gene encodes a melanoma-specific antigen recognized by tumor infiltrating lymphocytes. J Exp Med.

[22] Brandle D, Brasseur F, Weynants P, Boon T (1996). Van den Eynde B. A mutated HLA-A2 molecule recognized by autologous cytotoxic T lymphocytes on a human renal cell carcinoma. J Exp Med.

[23] Huang J, El-Gamil M, Dudley ME, Li YF, Rosenberg SA, Robbins PF (2004). T cells associated with tumor regression recognize frameshifted products of the CDKN2A tumor suppressor gene locus and a mutated HLA class I gene product. J Immunol.

[24] Lennerz V, Fatho M, Gentilini C, Frye RA, Lifke A, Ferel D, Wolfel C, Huber C, Wolfel T (2005). The response of autologous T cells to a human melanoma is dominated by mutated neoantigens. Proc Natl Acad Sci U S A.

[25] Zhou J, Dudley ME, Rosenberg SA, Robbins PF (2005). Persistence of multiple tumor-specific T-cell clones is associated with complete tumor regression in a melanoma patient receiving adoptive cell transfer therapy. J Immunother.

[26] Matsushita H, Vesely MD, Koboldt DC, Rickert CG, Uppaluri R, Magrini VJ, Arthur CD, White JM, Chen YS, Shea LK, Hundal J, Wendl MC, Demeter R, Wylie T, Allison JP, Smyth MJ, Old LJ, Mardis ER, Schreiber RD (2012). Cancer exome analysis reveals a T-cell-dependent mechanism of cancer immunoediting. Nature.

[27] Castle JC, Kreiter S, Diekmann J, Lower M, van de Roemer N, de Graaf J, Selmi A, Diken M, Boegel S, Paret C, Koslowski M, Kuhn AN, Britten CM, Huber C, Tureci O, Sahin U (2012). Exploiting the mutanome for tumor vaccination. Cancer Res.

[28] Robbins PF, Lu YC, El-Gamil M, Li YF, Gross C, Gartner J, Lin JC, Teer JK, Cliften P, Tycksen E, Samuels Y, Rosenberg SA (2013). Mining exomic sequencing data to identify mutated antigens recognized by adoptively transferred tumor-reactive T cells. Nat Med.

[29] Linnemann C, van Buuren MM, Bies L, Verdegaal EM, Schotte R, Calis JJ, Behjati S, Velds A, Hilkmann H, Atmioui DE, Visser M, Stratton MR, Haanen JB, Spits H, van der Burg SH, Schumacher TN (2015). High-throughput epitope discovery reveals frequent recognition of neo-antigens by CD4+ T cells in human melanoma. Nat Med.

[30] Tran E, Robbins PF, Lu YC, Prickett TD, Gartner JJ, Jia L, Pasetto A, Zheng Z, Ray S, Groh EM, Kriley IR, Rosenberg SA (2016). T-cell transfer therapy targeting mutant KRAS in cancer. N Engl J Med.

[31] Verdegaal EM, de Miranda NF, Visser M, Harryvan T, van Buuren MM, Andersen RS, Hadrup SR, van der Minne CE, Schotte R, Spits H, Haanen JB, Kapiteijn EH, Schumacher TN, van der Burg SH (2016). Neoantigen landscape dynamics during human melanoma-T cell interactions. Nature.

[32] Stevanovic S, Pasetto A, Helman SR, Gartner JJ, Prickett TD, Howie B, Robins HS, Robbins PF, Klebanoff CA, Rosenberg SA, Hinrichs CS (2017). Landscape of immunogenic tumor antigens in successful immunotherapy of virally induced epithelial cancer. Science.

[33] Rizvi NA, Hellmann MD, Snyder A, Kvistborg P, Makarov V, Havel JJ, Lee W, Yuan J, Wong P, Ho TS, Miller ML, Rekhtman N, Moreira AL, Ibrahim F, Bruggeman C, Gasmi B, Zappasodi R, Maeda Y, Sander C, Garon EB, Merghoub T, Wolchok JD, Schumacher TN, Chan TA (2015). Cancer immunology. Mutational landscape determines sensitivity to PD-1 blockade in non-small cell lung cancer. Science.

[34] Chen DS, Mellman I (2013). Oncology meets immunology: the cancer-immunity cycle. Immunity.

[35] Chen DS, Mellman I (2017). Elements of cancer immunity and the cancer-immune set point. Nature.

[36] Snyder A, Makarov V, Merghoub T, Yuan J, Zaretsky JM, Desrichard A, Walsh LA, Postow MA, Wong P, Ho TS, Hollmann TJ, Bruggeman C, Kannan K, Li Y, Elipenahli C, Liu C, Harbison CT, Wang L, Ribas A, Wolchok JD, Chan TA (2014). Genetic basis for clinical response to CTLA-4 blockade in melanoma. N Engl J Med.

[37] Van Allen EM, Miao D, Schilling B, Shukla SA, Blank C, Zimmer L, Sucker A, Hillen U, Foppen MHG, Goldinger SM, Utikal J, Hassel JC, Weide B, Kaehler KC, Loquai C, Mohr P, Gutzmer R, Dummer R, Gabriel S, Wu CJ, Schadendorf D, Garraway LA (2015). Genomic correlates of response to CTLA-4 blockade in metastatic melanoma. Science.

[38] Yarchoan M, Hopkins A, Jaffee EM (2017). Tumor mutational burden and response rate to PD-1 inhibition. N Engl J Med.

[39] McGranahan N, Furness AJ, Rosenthal R, Ramskov S, Lyngaa R, Saini SK, Jamal-Hanjani M, Wilson GA, Birkbak NJ, Hiley CT, Watkins TB, Shafi S, Murugaesu N, Mitter R, Akarca AU, Linares J, Marafioti T, Henry JY, Van Allen EM, Miao D, Schilling B, Schadendorf D, Garraway LA, Makarov V, Rizvi NA, Snyder A, Hellmann MD, Merghoub T, Wolchok JD, Shukla SA, Wu CJ, Peggs KS, Chan TA, Hadrup SR, Quezada SA, Swanton C (2016). Clonal neoantigens elicit T cell immunoreactivity and sensitivity to immune checkpoint blockade. Science.

[40] Duan F, Duitama J, Al Seesi S, Ayres CM, Corcelli SA, Pawashe AP, Blanchard T, McMahon D, Sidney J, Sette A, Baker BM, Mandoiu II, Srivastava PK (2014). Genomic and bioinformatic profiling of mutational neoepitopes reveals new rules to predict anticancer immunogenicity. J Exp Med.

[41] Fritsch EF, Rajasagi M, Ott PA, Brusic V, Hacohen N, Wu CJ (2014). HLA-binding properties of tumor neoepitopes in humans. Cancer Immunol Res.

[42] Milicic A, Price DA, Zimbwa P, Booth BL, Brown HL, Easterbrook PJ, Olsen K, Robinson N, Gileadi U, Sewell AK, Cerundolo V, Phillips RE (2005). CD8+ T cell epitope-flanking mutations disrupt proteasomal processing of HIV-1 Nef. J Immunol.

[43] Eisenlohr LC, Yewdell JW, Bennink JR (1992). Flanking sequences influence the presentation of an endogenously synthesized peptide to cytotoxic T lymphocytes. J Exp Med.

[44] Hutchison S, Pritchard AL (2018). Identifying neoantigens for use in immunotherapy. Mamm Genome.

[45] Pritchard AL, Burel JG, Neller MA, Hayward NK, Lopez JA, Fatho M, Lennerz V, Wolfel T, Schmidt CW (2015). Exome sequencing to predict neoantigens in melanoma. Cancer Immunol Res.

[46] Gubin MM, Artyomov MN, Mardis ER, Schreiber RD (2015). Tumor neoantigens: building a framework for personalized cancer immunotherapy. J Clin Invest.

[47] Jurtz V, Paul S, Andreatta M, Marcatili P, Peters B, Nielsen M (2017). NetMHCpan-4.0: Improved peptide-MHC Class I interaction predictions integrating eluted ligand and peptide binding affinity data. J Immunol.

[48] Jensen KK, Andreatta M, Marcatili P, Buus S, Greenbaum JA, Yan Z, Sette A, Peters B, Nielsen M (2018). Improved methods for predicting peptide binding affinity to MHC class II molecules. Immunology.

[49] O'Donnell TJ, Rubinsteyn A, Bonsack M, Riemer AB, Laserson U, Hammerbacher J (2018). MHCflurry: open-source Class I MHC binding affinity prediction. Cell Syst.

[50] Han Y, Kim D (2017). Deep convolutional neural networks for pan-specific peptide-MHC class I binding prediction. BMC Bioinformatics.

[51] Alvaro-Benito M, Morrison E, Abualrous ET, Kuropka B, Freund C (2018). Quantification of HLA-DM-dependent major histocompatibility complex of Class II immunopeptidomes by the peptide landscape antigenic epitope alignment utility. Front Immunol.

[52] Stranzl T, Larsen MV, Lundegaard C, Nielsen M (2010). NetCTLpan: pan-specific MHC class I pathway epitope predictions. Immunogenetics.

[53] Bjerregaard AM, Nielsen M, Jurtz V, Barra CM, Hadrup SR, Szallasi Z, Eklund AC (2017). An analysis of natural T cell responses to predicted tumor neoepitopes. Front Immunol.

[54] Martins Joana, Magalhães Carlos, Rocha Miguel, Osório Nuno S (2019). Machine Learning-Enhanced T Cell Neoepitope Discovery for Immunotherapy Design. Cancer Informatics.

[55] Vita R, Overton JA, Greenbaum JA, Ponomarenko J, Clark JD, Cantrell JR, Wheeler DK, Gabbard JL, Hix D, Sette A, Peters B (2015). The immune epitope database (IEDB) 3.0. Nucleic Acids Res.

[56] Yadav M, Jhunjhunwala S, Phung QT, Lupardus P, Tanguay J, Bumbaca S, Franci C, Cheung TK, Fritsche J, Weinschenk T, Modrusan Z, Mellman I, Lill JR, Delamarre L (2014). Predicting immunogenic tumour mutations by combining mass spectrometry and exome sequencing. Nature.

[57] Freudenmann LK, Marcu A, Stevanovic S (2018). Mapping the tumour human leukocyte antigen (HLA) ligandome by mass spectrometry. Immunology.

[58] Abelin JG, Keskin DB, Sarkizova S, Hartigan CR, Zhang W, Sidney J, Stevens J, Lane W, Zhang GL, Eisenhaure TM, Clauser KR, Hacohen N, Rooney MS, Carr SA, Wu CJ (2017). Mass spectrometry profiling of HLA-associated peptidomes in mono-allelic cells enables more accurate epitope prediction. Immunity.

[59] Bulik-Sullivan Brendan, Busby Jennifer, Palmer Christine D, Davis Matthew J, Murphy Tyler, Clark Andrew, Busby Michele, Duke Fujiko, Yang Aaron, Young Lauren, Ojo Noelle C, Caldwell Kamilah, Abhyankar Jesse, Boucher Thomas, Hart Meghan G, Makarov Vladimir, De Montpreville Vincent Thomas, Mercier Olaf, Chan Timothy A, Scagliotti Giorgio, Bironzo Paolo, Novello Silvia, Karachaliou Niki, Rosell Rafael, Anderson Ian, Gabrail Nashat, Hrom John, Limvarapuss Chainarong, Choquette Karin, Spira Alexander, Rousseau Raphael, Voong Cynthia, Rizvi Naiyer A, Fadel Elie, Frattini Mark, Jooss Karin, Skoberne Mojca, Francis Joshua, Yelensky Roman (2018). Deep learning using tumor HLA peptide mass spectrometry datasets improves neoantigen identification. Nature Biotechnology.

[60] Bjerregaard AM, Nielsen M, Hadrup SR, Szallasi Z, Eklund AC (2017). MuPeXI: prediction of neo-epitopes from tumor sequencing data. Cancer Immunol Immunother.

[61] Chowell D, Krishna S, Becker PD, Cocita C, Shu J, Tan X, Greenberg PD, Klavinskis LS, Blattman JN, Anderson KS (2015). TCR contact residue hydrophobicity is a hallmark of immunogenic CD8+ T cell epitopes. Proc Natl Acad Sci U S A.

[62] Wilson EA, Anderson KS (2018). Lost in the crowd: identifying targetable MHC class I neoepitopes for cancer immunotherapy. Expert Rev Proteomics.

[63] Kim S, Kim HS, Kim E, Lee MG, Shin EC, Paik S, Kim S (2018). Neopepsee: accurate genome-level prediction of neoantigens by harnessing sequence and amino acid immunogenicity information. Ann Oncol.

[64] Schreiber RD, Old LJ, Smyth MJ (2011). Cancer immunoediting: integrating immunity’s roles in cancer suppression and promotion. Science.

[65] DuPage M, Cheung AF, Mazumdar C, Winslow MM, Bronson R, Schmidt LM, Crowley D, Chen J, Jacks T (2011). Endogenous T cell responses to antigens expressed in lung adenocarcinomas delay malignant tumor progression. Cancer Cell.

[66] DuPage M, Mazumdar C, Schmidt LM, Cheung AF, Jacks T (2012). Expression of tumour-specific antigens underlies cancer immunoediting. Nature.

[67] von Boehmer L, Mattle M, Bode P, Landshammer A, Schafer C, Nuber N, Ritter G, Old L, Moch H, Schafer N, Jager E, Knuth A, van den Broek M (2013). NY-ESO-1-specific immunological pressure and escape in a patient with metastatic melanoma. Cancer Immun.

[68] Mittal D, Gubin MM, Schreiber RD, Smyth MJ (2014). New insights into cancer immunoediting and its three component phases--elimination, equilibrium and escape. Curr Opin Immunol.

[69] Kim R, Emi M, Tanabe K (2007). Cancer immunoediting from immune surveillance to immune escape. Immunology.

[70] Vesely MD, Schreiber RD (2013). Cancer immunoediting: antigens, mechanisms, and implications to cancer immunotherapy. Ann N Y Acad Sci.

[71] van Rooij N, van Buuren MM, Philips D, Velds A, Toebes M, Heemskerk B, van Dijk LJ, Behjati S, Hilkmann H, El Atmioui D, Nieuwland M, Stratton MR, Kerkhoven RM, Kesmir C, Haanen JB, Kvistborg P, Schumacher TN (2013). Tumor exome analysis reveals neoantigen-specific T-cell reactivity in an ipilimumab-responsive melanoma. J Clin Oncol.

[72] Gerlinger M, Rowan AJ, Horswell S, Math M, Larkin J, Endesfelder D, Gronroos E, Martinez P, Matthews N, Stewart A, Tarpey P, Varela I, Phillimore B, Begum S, McDonald NQ, Butler A, Jones D, Raine K, Latimer C, Santos CR, Nohadani M, Eklund AC, Spencer-Dene B, Clark G, Pickering L, Stamp G, Gore M, Szallasi Z, Downward J, Futreal PA, Swanton C (2012). Intratumor heterogeneity and branched evolution revealed by multiregion sequencing. N Engl J Med.

[73] Anagnostou V, Smith KN, Forde PM, Niknafs N, Bhattacharya R, White J, Zhang T, Adleff V, Phallen J, Wali N, Hruban C, Guthrie VB, Rodgers K, Naidoo J, Kang H, Sharfman W, Georgiades C, Verde F, Illei P, Li QK, Gabrielson E, Brock MV, Zahnow CA, Baylin SB, Scharpf RB, Brahmer JR, Karchin R, Pardoll DM, Velculescu VE (2017). Evolution of neoantigen landscape during immune checkpoint blockade in non-small cell lung cancer. Cancer Discov.

[74] Angelova M, Mlecnik B, Vasaturo A, Bindea G, Fredriksen T, Lafontaine L, Buttard B, Morgand E, Bruni D, Jouret-Mourin A, Hubert C, Kartheuser A, Humblet Y, Ceccarelli M, Syed N, Marincola FM, Bedognetti D, Van den Eynde M, Galon J (2018). Evolution of metastases in space and time under immune selection. Cell.

[75] Davidson G, Coassolo S, Kieny A, Ennen M, Pencreach E, Malouf GG, Lipsker D, Davidson I (2019). Dynamic evolution of clonal composition and neoantigen landscape in recurrent metastatic melanoma with a rare combination of driver mutations. J Invest Dermatol.

[76] Nejo T, Matsushita H, Karasaki T, Nomura M, Saito K, Tanaka S, Takayanagi S, Hana T, Takahashi S, Kitagawa Y, Koike T, Kobayashi Y, Nagae G, Yamamoto S, Ueda H, Tatsuno K, Narita Y, Nagane M, Ueki K, Nishikawa R, Aburatani H, Mukasa A, Saito N, Kakimi K (2019). Reduced neoantigen expression revealed by longitudinal multiomics as a possible immune evasion mechanism in glioma. Cancer Immunol Res.

[77] Rosenthal R, Cadieux EL, Salgado R, Bakir MA, Moore DA, Hiley CT, Lund T, Tanic M, Reading JL, Joshi K, Henry JY, Ghorani E, Wilson GA, Birkbak NJ, Jamal-Hanjani M, Veeriah S, Szallasi Z, Loi S, Hellmann MD, Feber A, Chain B, Herrero J, Quezada SA, Demeulemeester J, Van Loo P, Beck S, McGranahan N, Swanton C, consortium TR (2019). Neoantigen-directed immune escape in lung cancer evolution. Nature.

[78] Zhang J, Caruso FP, Sa JK, Justesen S, Nam DH, Sims P, Ceccarelli M, Lasorella A, Iavarone A (2019). The combination of neoantigen quality and T lymphocyte infiltrates identifies glioblastomas with the longest survival. Commun Biol.

[79] Balachandran Vinod P., Łuksza Marta, Zhao Julia N., Makarov Vladimir, Moral John Alec, Remark Romain, Herbst Brian, Askan Gokce, Bhanot Umesh, Senbabaoglu Yasin, Wells Daniel K., Cary Charles Ian Ormsby, Grbovic-Huezo Olivera, Attiyeh Marc, Medina Benjamin, Zhang Jennifer, Loo Jennifer, Saglimbeni Joseph, Abu-Akeel Mohsen, Zappasodi Roberta, Riaz Nadeem, Smoragiewicz Martin, Kelley Z. Larkin, Basturk Olca, Gönen Mithat, Levine Arnold J., Allen Peter J., Fearon Douglas T., Merad Miriam, Gnjatic Sacha, Iacobuzio-Donahue Christine A., Wolchok Jedd D., DeMatteo Ronald P., Chan Timothy A., Greenbaum Benjamin D., Merghoub Taha, Leach Steven D. (2017). Identification of unique neoantigen qualities in long-term survivors of pancreatic cancer. Nature.

[80] Dunn J, Rao S (2017). Epigenetics and immunotherapy: the current state of play. Mol Immunol.

[81] Garrido F, Ruiz-Cabello F, Aptsiauri N (2017). Rejection versus escape: the tumor MHC dilemma. Cancer Immunol Immunother.

[82] Oh DY, Fong L (2018). Immunity in the time of metastases. Immunity.

[83] Popovic A, Jaffee EM, Zaidi N (2018). Emerging strategies for combination checkpoint modulators in cancer immunotherapy. J Clin Invest.

[84] Melero I, Gaudernack G, Gerritsen W, Huber C, Parmiani G, Scholl S, Thatcher N, Wagstaff J, Zielinski C, Faulkner I, Mellstedt H (2014). Therapeutic vaccines for cancer: an overview of clinical trials. Nat Rev Clin Oncol.

[85] Yarchoan M, Johnson BA, Lutz ER, Laheru DA, Jaffee EM (2017). Targeting neoantigens to augment antitumour immunity. Nat Rev Cancer.

[86] Kreiter S, Vormehr M, van de Roemer N, Diken M, Lower M, Diekmann J, Boegel S, Schrors B, Vascotto F, Castle JC, Tadmor AD, Schoenberger SP, Huber C, Tureci O, Sahin U (2015). Mutant MHC class II epitopes drive therapeutic immune responses to cancer. Nature.

[87] Aurisicchio L, Salvatori E, Lione L, Bandini S, Pallocca M, Maggio R, Fanciulli M, De Nicola F, Goeman F, Ciliberto G, Conforti A, Luberto L, Palombo F (2019). Poly-specific neoantigen-targeted cancer vaccines delay patient derived tumor growth. J Exp Clin Cancer Res.

[88] Duperret EK, Perales-Puchalt A, Stoltz R, HH G, Mandloi N, Barlow J, Chaudhuri A, Sardesai NY, Weiner DB (2019). A synthetic DNA, multi-neoantigen vaccine drives predominately MHC Class I CD8(+) T-cell responses, impacting tumor challenge. Cancer Immunol Res.

[89] Kuai R, Ochyl LJ, Bahjat KS, Schwendeman A, Moon JJ (2017). Designer vaccine nanodiscs for personalized cancer immunotherapy. Nat Mater.

[90] Forghanifard MM, Gholamin M, Moaven O, Farshchian M, Ghahraman M, Aledavood A, Abbaszadegan MR (2014). Neoantigen in esophageal squamous cell carcinoma for dendritic cell-based cancer vaccine development. Med Oncol.

[91] Gubin MM, Zhang X, Schuster H, Caron E, Ward JP, Noguchi T, Ivanova Y, Hundal J, Arthur CD, Krebber WJ, Mulder GE, Toebes M, Vesely MD, Lam SS, Korman AJ, Allison JP, Freeman GJ, Sharpe AH, Pearce EL, Schumacher TN, Aebersold R, Rammensee HG, Melief CJ, Mardis ER, Gillanders WE, Artyomov MN, Schreiber RD (2014). Checkpoint blockade cancer immunotherapy targets tumour-specific mutant antigens. Nature.

[92] Schumacher T, Bunse L, Pusch S, Sahm F, Wiestler B, Quandt J, Menn O, Osswald M, Oezen I, Ott M, Keil M, Balss J, Rauschenbach K, Grabowska AK, Vogler I, Diekmann J, Trautwein N, Eichmuller SB, Okun J, Stevanovic S, Riemer AB, Sahin U, Friese MA, Beckhove P, von Deimling A, Wick W, Platten M (2014). A vaccine targeting mutant IDH1 induces antitumour immunity. Nature.

[93] Carreno BM, Magrini V, Becker-Hapak M, Kaabinejadian S, Hundal J, Petti AA, Ly A, Lie WR, Hildebrand WH, Mardis ER, Linette GP (2015). Cancer immunotherapy. A dendritic cell vaccine increases the breadth and diversity of melanoma neoantigen-specific T cells. Science.

[94] Hilf N, Kuttruff-Coqui S, Frenzel K, Bukur V, Stevanovic S, Gouttefangeas C, Platten M, Tabatabai G, Dutoit V, van der Burg SH, Thor Straten P, Martinez-Ricarte F, Ponsati B, Okada H, Lassen U, Admon A, Ottensmeier CH, Ulges A, Kreiter S, von Deimling A, Skardelly M, Migliorini D, Kroep JR, Idorn M, Rodon J, Piro J, Poulsen HS, Shraibman B, McCann K, Mendrzyk R, Lower M, Stieglbauer M, Britten CM, Capper D, Welters MJP, Sahuquillo J, Kiesel K, Derhovanessian E, Rusch E, Bunse L, Song C, Heesch S, Wagner C, Kemmer-Bruck A, Ludwig J, Castle JC, Schoor O, Tadmor AD, Green E, Fritsche J, Meyer M, Pawlowski N, Dorner S, Hoffgaard F, Rossler B, Maurer D, Weinschenk T, Reinhardt C, Huber C, Rammensee HG, Singh-Jasuja H, Sahin U, Dietrich PY, Wick W (2019). Actively personalized vaccination trial for newly diagnosed glioblastoma. Nature.

[95] Keskin DB, Anandappa AJ, Sun J, Tirosh I, Mathewson ND, Li S, Oliveira G, Giobbie-Hurder A, Felt K, Gjini E, Shukla SA, Hu Z, Li L, Le PM, Allesoe RL, Richman AR, Kowalczyk MS, Abdelrahman S, Geduldig JE, Charbonneau S, Pelton K, Iorgulescu JB, Elagina L, Zhang W, Olive O, McCluskey C, Olsen LR, Stevens J, Lane WJ, Salazar AM, Daley H, Wen PY, Chiocca EA, Harden M, Lennon NJ, Gabriel S, Getz G, Lander ES, Regev A, Ritz J, Neuberg D, Rodig SJ, Ligon KL, Suva ML, Wucherpfennig KW, Hacohen N, Fritsch EF, Livak KJ, Ott PA, Wu CJ, Reardon DA (2019). Neoantigen vaccine generates intratumoral T cell responses in phase Ib glioblastoma trial. Nature.

[96] Ott PA, Hu Z, Keskin DB, Shukla SA, Sun J, Bozym DJ, Zhang W, Luoma A, Giobbie-Hurder A, Peter L, Chen C, Olive O, Carter TA, Li S, Lieb DJ, Eisenhaure T, Gjini E, Stevens J, Lane WJ, Javeri I, Nellaiappan K, Salazar AM, Daley H, Seaman M, Buchbinder EI, Yoon CH, Harden M, Lennon N, Gabriel S, Rodig SJ, Barouch DH, Aster JC, Getz G, Wucherpfennig K, Neuberg D, Ritz J, Lander ES, Fritsch EF, Hacohen N, Wu CJ (2017). An immunogenic personal neoantigen vaccine for patients with melanoma. Nature.

[97] Sahin U, Derhovanessian E, Miller M, Kloke BP, Simon P, Lower M, Bukur V, Tadmor AD, Luxemburger U, Schrors B, Omokoko T, Vormehr M, Albrecht C, Paruzynski A, Kuhn AN, Buck J, Heesch S, Schreeb KH, Muller F, Ortseifer I, Vogler I, Godehardt E, Attig S, Rae R, Breitkreuz A, Tolliver C, Suchan M, Martic G, Hohberger A, Sorn P, Diekmann J, Ciesla J, Waksmann O, Bruck AK, Witt M, Zillgen M, Rothermel A, Kasemann B, Langer D, Bolte S, Diken M, Kreiter S, Nemecek R, Gebhardt C, Grabbe S, Holler C, Utikal J, Huber C, Loquai C, Tureci O (2017). Personalized RNA mutanome vaccines mobilize poly-specific therapeutic immunity against cancer. Nature.

[98] Kranz LM, Diken M, Haas H, Kreiter S, Loquai C, Reuter KC, Meng M, Fritz D, Vascotto F, Hefesha H, Grunwitz C, Vormehr M, Husemann Y, Selmi A, Kuhn AN, Buck J, Derhovanessian E, Rae R, Attig S, Diekmann J, Jabulowsky RA, Heesch S, Hassel J, Langguth P, Grabbe S, Huber C, Tureci O, Sahin U (2016). Systemic RNA delivery to dendritic cells exploits antiviral defence for cancer immunotherapy. Nature.

[99] Tran E, Ahmadzadeh M, Lu YC, Gros A, Turcotte S, Robbins PF, Gartner JJ, Zheng Z, Li YF, Ray S, Wunderlich JR, Somerville RP, Rosenberg SA (2015). Immunogenicity of somatic mutations in human gastrointestinal cancers. Science.

[100] Yang W, Lee KW, Srivastava RM, Kuo F, Krishna C, Chowell D, Makarov V, Hoen D, Dalin MG, Wexler L, Ghossein R, Katabi N, Nadeem Z, Cohen MA, Tian SK, Robine N, Arora K, Geiger H, Agius P, Bouvier N, Huberman K, Vanness K, Havel JJ, Sims JS, Samstein RM, Mandal R, Tepe J, Ganly I, Ho AL, Riaz N, Wong RJ, Shukla N, Chan TA, Morris LGT (2019). Immunogenic neoantigens derived from gene fusions stimulate T cell responses. Nat Med.

[101] Ren L, Leisegang M, Deng B, Matsuda T, Kiyotani K, Kato T, Harada M, Park JH, Saloura V, Seiwert T, Vokes E, Agrawal N, Nakamura Y (2019). Identification of neoantigen-specific T cells and their targets: implications for immunotherapy of head and neck squamous cell carcinoma. Oncoimmunology.

[102] Veatch JR, Jesernig BL, Kargl J, Fitzgibbon M, Lee SM, Baik C, Martins R, Houghton AM, Riddell SR. Endogenous CD4(+) T cells recognize neoantigens in lung cancer patients, including recurrent oncogenic KRAS and ERBB2 (Her2) driver mutations. Cancer Immunol Res. 2019. 10.1158/2326-6066.CIR-18-0402 PMC6584616 31043415

[103] Lo W, Parkhurst M, Robbins PF, Tran E, Lu YC, Jia L, Gartner JJ, Pasetto A, Deniger D, Malekzadeh P, Shelton TE, Prickett T, Ray S, Kivitz S, Paria BC, Kriley I, Schrump DS, Rosenberg SA (2019). Immunologic recognition of a shared p53 mutated neoantigen in a patient with metastatic colorectal cancer. Cancer Immunol Res.

[104] Zhang X, Kim S, Hundal J, Herndon JM, Li S, Petti AA, Soysal SD, Li L, McLellan MD, Hoog J, Primeau T, Myers N, Vickery TL, Sturmoski M, Hagemann IS, Miller CA, Ellis MJ, Mardis ER, Hansen T, Fleming TP, Goedegebuure SP, Gillanders WE (2017). Breast cancer neoantigens can induce CD8(+) T-cell responses and antitumor immunity. Cancer Immunol Res.

[105] Nelde A, Walz JS, Kowalewski DJ, Schuster H, Wolz OO, Peper JK, Cardona Gloria Y, Langerak AW, Muggen AF, Claus R, Bonzheim I, Fend F, Salih HR, Kanz L, Rammensee HG, Stevanovic S, Weber AN (2017). HLA class I-restricted MYD88 L265P-derived peptides as specific targets for lymphoma immunotherapy. Oncoimmunology.

[106] Wang QJ, Yu Z, Griffith K, Hanada K, Restifo NP, Yang JC (2016). Identification of T-cell receptors targeting KRAS-mutated human tumors. Cancer Immunol Res.

[107] Zacharakis N, Chinnasamy H, Black M, Xu H, Lu YC, Zheng Z, Pasetto A, Langhan M, Shelton T, Prickett T, Gartner J, Jia L, Trebska-McGowan K, Somerville RP, Robbins PF, Rosenberg SA, Goff SL, Feldman SA (2018). Immune recognition of somatic mutations leading to complete durable regression in metastatic breast cancer. Nat Med.

[108] Cafri G, Yossef R, Pasetto A, Deniger DC, Lu YC, Parkhurst M, Gartner JJ, Jia L, Ray S, Ngo LT, Jafferji M, Sachs A, Prickett T, Robbins PF, Rosenberg SA (2019). Memory T cells targeting oncogenic mutations detected in peripheral blood of epithelial cancer patients. Nat Commun.

[109] Malekzadeh P, Pasetto A, Robbins PF, Parkhurst MR, Paria BC, Jia L, Gartner JJ, Hill V, Yu Z, Restifo NP, Sachs A, Tran E, Lo W, Somerville RP, Rosenberg SA, Deniger DC (2019). Neoantigen screening identifies broad TP53 mutant immunogenicity in patients with epithelial cancers. J Clin Invest.

[110] Tran E, Turcotte S, Gros A, Robbins PF, Lu YC, Dudley ME, Wunderlich JR, Somerville RP, Hogan K, Hinrichs CS, Parkhurst MR, Yang JC, Rosenberg SA (2014). Cancer immunotherapy based on mutation-specific CD4+ T cells in a patient with epithelial cancer. Science.

[111] Chen F, Zou Z, Du J, Su S, Shao J, Meng F, Yang J, Xu Q, Ding N, Yang Y, Liu Q, Wang Q, Sun Z, Zhou S, Du S, Wei J, Liu B. Neoantigen identification strategies enable personalized immunotherapy in refractory solid tumors. J Clin Invest. 2019;130. 10.1172/JCI99538 PMC6486339 30835255

[112] Sahin U, Tureci O (2018). Personalized vaccines for cancer immunotherapy. Science.

[113] Hu Z, Ott PA, Wu CJ (2018). Towards personalized, tumour-specific, therapeutic vaccines for cancer. Nat Rev Immunol.

[114] Zitvogel L, Kepp O, Kroemer G (2010). Decoding cell death signals in inflammation and immunity. Cell.

[115] Beatty GL, Gladney WL (2015). Immune escape mechanisms as a guide for cancer immunotherapy. Clin Cancer Res.

[116] Zappasodi R, Merghoub T, Wolchok JD (2018). Emerging concepts for immune checkpoint blockade-based combination therapies. Cancer Cell.

[117] Patel SA, Minn AJ (2018). Combination cancer therapy with immune checkpoint blockade: mechanisms and strategies. Immunity.

[118] Wu J, Waxman DJ (2018). Immunogenic chemotherapy: dose and schedule dependence and combination with immunotherapy. Cancer Lett.

[119] Gotwals P, Cameron S, Cipolletta D, Cremasco V, Crystal A, Hewes B, Mueller B, Quaratino S, Sabatos-Peyton C, Petruzzelli L, Engelman JA, Dranoff G (2017). Prospects for combining targeted and conventional cancer therapy with immunotherapy. Nat Rev Cancer.

[120] Galon J, Bruni D (2019). Approaches to treat immune hot, altered and cold tumours with combination immunotherapies. Nat Rev Drug Discov.

[121] Alexandrov LB, Nik-Zainal S, Wedge DC, Aparicio SA, Behjati S, Biankin AV, Bignell GR, Bolli N, Borg A, Borresen-Dale AL, Boyault S, Burkhardt B, Butler AP, Caldas C, Davies HR, Desmedt C, Eils R, Eyfjord JE, Foekens JA, Greaves M, Hosoda F, Hutter B, Ilicic T, Imbeaud S, Imielinski M, Jager N, Jones DT, Jones D, Knappskog S, Kool M, Lakhani SR, Lopez-Otin C, Martin S, Munshi NC, Nakamura H, Northcott PA, Pajic M, Papaemmanuil E, Paradiso A, Pearson JV, Puente XS, Raine K, Ramakrishna M, Richardson AL, Richter J, Rosenstiel P, Schlesner M, Schumacher TN, Span PN, Teague JW, Totoki Y, Tutt AN, Valdes-Mas R, van Buuren MM, van ‘t Veer L, Vincent-Salomon A, Waddell N, Yates LR, Zucman-Rossi J, Futreal PA, McDermott U, Lichter P, Meyerson M, Grimmond SM, Siebert R, Campo E, Shibata T, Pfister SM, Campbell PJ, Stratton MR, Australian Pancreatic Cancer Genome I, Consortium IBC, Consortium IM-S, PedBrain I (2013). Signatures of mutational processes in human cancer. Nature.

[122] Chalmers ZR, Connelly CF, Fabrizio D, Gay L, Ali SM, Ennis R, Schrock A, Campbell B, Shlien A, Chmielecki J, Huang F, He Y, Sun J, Tabori U, Kennedy M, Lieber DS, Roels S, White J, Otto GA, Ross JS, Garraway L, Miller VA, Stephens PJ, Frampton GM (2017). Analysis of 100,000 human cancer genomes reveals the landscape of tumor mutational burden. Genome Med.

[123] Le DT, Durham JN, Smith KN, Wang H, Bartlett BR, Aulakh LK, Lu S, Kemberling H, Wilt C, Luber BS, Wong F, Azad NS, Rucki AA, Laheru D, Donehower R, Zaheer A, Fisher GA, Crocenzi TS, Lee JJ, Greten TF, Duffy AG, Ciombor KK, Eyring AD, Lam BH, Joe A, Kang SP, Holdhoff M, Danilova L, Cope L, Meyer C, Zhou S, Goldberg RM, Armstrong DK, Bever KM, Fader AN, Taube J, Housseau F, Spetzler D, Xiao N, Pardoll DM, Papadopoulos N, Kinzler KW, Eshleman JR, Vogelstein B, Anders RA, Diaz LA (2017). Mismatch repair deficiency predicts response of solid tumors to PD-1 blockade. Science.

[124] Luksza M, Riaz N, Makarov V, Balachandran VP, Hellmann MD, Solovyov A, Rizvi NA, Merghoub T, Levine AJ, Chan TA, Wolchok JD, Greenbaum BD (2017). A neoantigen fitness model predicts tumour response to checkpoint blockade immunotherapy. Nature.

